# Publisher Correction: Linking solar minimum, space weather, and night sky brightness

**DOI:** 10.1038/s41598-023-36648-6

**Published:** 2023-06-13

**Authors:** Albert D. Grauer, Patricia A. Grauer

**Affiliations:** 1 Catalina Sky Survey, Lunar and Planetary Laboratory, University of Arizona, Tucson, USA; 2 Cosmic Campground International Dark Sky Sanctuary, Alma, NM USA; 3 PO Box 2143, Silver City, NM 88062 USA

Correction to: *Scientific Reports* 10.1038/s41598-021-02365-1, published online 13 December 2021

The original version of this Article contained an error in Reference 9, which was incorrectly given as:

Alarcon, M. R., Serra-Ricart, M., Lemes-Perera, A. & Mallorquın, M. Natural Night Sky Brightness during solar minimum. *Astron. J.* **1**, 1(2021).

The correct reference is listed below:

Alarcon, M. R., Serra-Ricart, M., Lemes-Perera, S., & Mallorquín, M. Natural night sky brightness during solar minimum. *Astron. J.*, **162**(1), 25. 10.3847/1538-3881/abfdaa (2021).

The original Article has been corrected.

